# Dna2 Responds to Endogenous and Exogenous Replication Stress in *Drosophila melanogaster*

**DOI:** 10.3390/genes16101133

**Published:** 2025-09-25

**Authors:** Ivan Rivera, Sabah Shammari, Hamiya Sohail, Christian Villegas, Zoha Wasim, Sze Hang Ip, Vada Becker, Kathryn P. Kohl, Eric P. Stoffregen, Christina I. Swanson, Elyse Bolterstein

**Affiliations:** 1 Biology Department, Northeastern Illinois University, Chicago, IL 60625, USA; 2 Biology Department, Arcadia University, Glenside, PA 19038, USAswansonc@arcadia.edu (C.I.S.); 3 Biology Department, Winthrop University, Rock Hill, SC 29733, USA; kohlk@winthrop.edu; 4 Physical, Life, Movement & Sport Sciences Division, Lewis-Clark State College, Lewiston, ID 83501, USA; epstoffregen@lcsc.edu

**Keywords:** Dna2, Drosophila, DNA replication, replication stress, DNA repair, mutagen sensitivity, oogenesis, embryogenesis, genome stability

## Abstract

**Background/Objectives**: DNA2 is a conserved nuclease–helicase that plays a crucial role in DNA replication and repair by responding to replication stress. Previous studies have established the role of DNA2 in Okazaki fragment processing, the recovery of stalled replication forks, and double-strand break repair. This study investigates the role of *Drosophila melanogaster* Dna2 in response to exogenous DNA damage and replication stress as well as during developmental stages involving intensive DNA replication. **Methods**: We used the *Drosophila* mutant alleles, *Dna2^D1^* and *Dna2^D2^*, which differ in the presence of the helicase 1A domain, to assess sensitivity to mutagens that cause various types of replication stress and DNA damage. We examined reproductive fitness through Mendelian ratio calculations, fecundity, egg viability assays, and assessed DNA damage via immunostaining of ovarian germaria. Lifespan assays were also conducted to examine adult survival. **Results**: *Dna2* mutants demonstrated significant sensitivity to replication stress induced by MMS, hydroxyurea, topotecan, and nitrogen mustard. *Dna2^lS/S1^* mutants exhibited higher survival than *Dna2^lS/D2^* upon exposure to topotecan and bleomycin, suggesting a possible helicase-specific role in damage response. Mutants exhibited decreased fecundity, reduced egg viability, and elevated DNA damage in mitotically active germline cells. Adult lifespan was not reduced in *Dna2* mutants, implying potential compensatory stress-response mechanisms. **Conclusions**: Our findings support a requirement of Dna2 in managing replication stress during critical developmental phases in *Drosophila*. These insights clarify the nuanced contributions of the nuclease and helicase domains of DNA2, suggesting potential domain-specific functions in genomic stability and repair mechanisms. This work provides a foundation that will enable future researchers to further dissect the complex roles of DNA2 in replication and repair pathways.

## 1. Introduction

Cells are continuously challenged by a variety of DNA damage arising from both endogenous sources, such as reactive oxygen species and replication errors, and exogenous sources like UV light and chemical mutagens. To maintain genomic integrity and ensure accurate transmission of genetic information, cells rely on DNA repair proteins that detect, process, and resolve DNA damage [1]. Failures in repair pathways are linked to a wide range of diseases, including cancer, neurodegeneration, and developmental disorders [2]. Because of their potential in treating diseases, it is important to develop models in which individual DNA repair proteins are characterized for further research.

The DNA2 protein is an essential and conserved nuclease–helicase involved in several DNA repair and replication pathways. A primary role of DNA2 is the removal of 5′ RNA-DNA flaps from Okazaki fragments during lagging strand synthesis in coordination with FEN1 [3]. In the absence of DNA2, excess single-stranded DNA can trigger DNA damage checkpoints [3,4]. Another well-known role of DNA2 is homologous recombination (HR)-mediated recovery or restart of stalled replication forks in both HR-dependent and HR-independent pathways [4,5,6,7]. DNA2 has also been shown to participate in end-resection during double-strand break (DSB) repair, specifically in replicating cells [3,8,9,10]; however, this is considered a secondary role to the involvement of DNA2 in replication fork recovery and restart. DNA2 overexpression has been shown to positively correlate with disease outcomes in several human cancers, leading to its exploration as a possible therapeutic target [6,10]. DNA2’s roles in replication and repair are accomplished by its two main enzymatic domains: a structure-specific nuclease, which plays the dominant role in DNA replication and repair [11,12] and two DNA-activated helicase/ATPases that shape a tunnel-like configuration that selectively guides single-stranded DNA to the nuclease active site [13].

*Dna2* in *Drosophila melanogaster* (formerly *mus109*) is an X-linked gene that shows high protein sequence homology with DNA2 in humans, mouse, and *Caenorhabditis elegans,* including the presence of a nuclease domain and two helicase domains (helicase 1A and 2A) connected by a stalk protein [14]. All commercially available *Dna2* mutant alleles (*Dna2^lS^, Dna2^D1^,* and *Dna2^D2^*) are hypersensitive to the alkylating agent methyl methanesulfonate (MMS), nitrogen mustard, and ɣ-radiation [14,15,16,17], which are known to cause bulky DNA adducts [18], DNA crosslinks [19], and DNA strand breaks through generation of reactive oxygen species [20], respectively. *Dna2* mutants’ sensitivity to these reagents suggest that Dna2 is heavily involved in DNA replication and repair. Additionally, there are slight, but non-significant differences in MMS sensitivity between the alleles, possibly due to their domain-specific mutations: *Dna2^lS^* contains a mutation in the nuclease domain and is homozygous lethal, while *Dna2^D1^* and *Dna2^D2^* contain mutations in the helicase domains and are considered hypomorphic (see Figure 3 in reference [14] for mutation map).

In this study, we further investigate the roles of *Drosophila* Dna2 in DNA replication and repair. We specifically focus on the alleles *Dna2^D1^* and *Dna2^D2^* to determine differential responses in the presence of the helicase 1A domain, which is present in *Dna2^D1^* but not *Dna2^D2^* [14]. Based on DNA2 function in other organisms, we predicted that *Dna2* mutants would show sensitivity to mutagens that cause replication stress and show deficiencies in developmental timepoints that are highly dependent upon rapid and accurate DNA replication. Because it is common for deficiencies in replication and repair to impact longevity [21], we also investigated the impact of the *Dna2* mutation on lifespan. Together, we show a connection linking the roles of Dna2 in replication to fly development and organismal physiology.

## 2. Materials and Methods

### 2.1. Fly Stocks and Maintenance

All fly stocks were maintained on solid cornmeal agar (BF Formula, Genesee Scientific, El Cajon, CA, USA) and kept at 25 °C under a 12 h:12 h light–dark cycle. Plates containing grape agar (Genesee Scientific) were used for egg collection. The following fly stocks were obtained from the Bloomington Drosophila Stock Center (BDSC): *Dna2^D1^* (BDSC# 2320), *Dna2^D2^* (BDSC# 2307), and *Dna2^lS^* (BDSC# 4168). All experiments used mutants that were created in *trans* with the *Dna2^lS^* (*Dna2^lS/D1^* and *Dna2^lS/D2^*) allele to mitigate genetic background effects and because the transheterozygotes have shown stronger sensitivity to MMS [14]. *w^1118^* flies served as the wildtype control when appropriate.

### 2.2. Mutagen Sensitivity Assays

Mutagen sensitivity assays were performed similarly to previous studies [14,22,23]. Briefly, Brood 1 (Day 0) was established by crossing 7–10 *Dna2^lS^*/*FM7c* females with 2–3 *Dna2^D1^* or *Dna2^D2^* males per vial. On day 3, the flies were flipped into new vials to establish Brood 2. On day 4, Brood 1 vials were treated with 250 μL of an aqueous mutagen solution: 20 mM Bleomycin (Thermo Fisher Scientific, Waltham, MA, USA), 50 μM Camptothecin (Thermo Fisher Scientific), 80 mM Hydroxyurea (Thermo Fisher Scientific), 0.00625–0.05% MMS (TCI America, Corona, CA, USA), 10% Nitrogen Mustard (Sigma Aldrich, St. Louis, MO, USA), 50 μM Potassium Bromate (Thermo Fisher Scientific), and 60 μM Topotecan (Apexbio Technology LLC, Houston, TX, USA). On day 5, the adult flies were discarded from Brood 2 vials, and on day 6, Brood 2 vials were mock-treated with 250 μL water. Adult offspring were collected every 2–3 days until day 18 (Brood 1) or day 21 (Brood 2) to ensure counting of only the first generation. Offspring were scored by sex and eye phenotype using the “bar-eye” phenotype on the *FM7c* balancer as an indicator of non-mutants and a “round-eye” phenotype (non-*FM7c*) for mutants. For each vial, relative survival was calculated by dividing the percentage of mutant flies in each treated vial (Brood 1) by the percentage of mutant flies in its corresponding control vial (Brood 2). Vials with fewer than 15 progeny in either Brood 1 or 2 were excluded from analysis. Statistical significance between *Dna2^lS/D1^* and *Dna2^lS/D2^* alleles was determined by unpaired t-test. Statistical significance for mutagen sensitivity was determined by unpaired t-tests comparing each mean relative survival with 100% survival, which would represent no sensitivity to the mutagen. Statistical analysis and graphing were performed using GraphPad Prism 10.5.0.

### 2.3. Mendelian Ratios

The offspring of the mock-treated controls in the mutagen sensitivity assays were used to determine Mendelian ratios, calculated by chi-square using the following classes of offspring: mutant females (*Dna2^lS/D1^* or *Dna2^lS/D2^*), non-mutant females (*Dna2^lS^/FM7c*), and non-mutant males (*FM7c/Y*). The mutant male class (*Dna2^lS^/Y*) was excluded from calculation because of the lethality of the *Dna2^lS^* allele. Calculations were performed using GraphPad Quickcalcs using an expected value of ⅓ for each class calculated. A small percentage (0.31–0.66%) of “round eye” (non-*FM7c*) males were observed. These males were presumed to be due to non-disjunction and not true *Dna2^lS^/Y* and were therefore omitted from calculations.

### 2.4. Fecundity

20–25 virgin female flies of the desired genotype (*w^1118^* or *Dna2^lS/D2^*) were combined with 10 *w^1118^* male flies in an embryo collection chamber (*Dna2^lS/D2^* was prioritized due to the stronger fertility phenotype). All flies were 0–2 days old when added to the chamber. Chambers were maintained at 25 °C and fed three times daily (9:00 a.m., 12:00 p.m., and 5:00 p.m.) during the assay period. We used the 9:00 a.m.–12:00 p.m. collection for our fecundity assay data as collections taken later in the day were found to be highly variable, even for wild-type flies. Assay data from days 3–9 were used for data analysis. The number of eggs laid was divided by the number of females present and by the length of the collection (3 h) to calculate the average number of eggs laid per female per hour. Statistical analysis via unpaired *t*-test and graphing were performed using GraphPad Prism 10.5.0.

### 2.5. Egg Viability

Embryo chambers were set up with 0–2 day old females of the desired genotype (*w^1118^*, *Dna2^lS/D1^*, or *Dna2^lS/D2^*) and *w^1118^* males. Collections began 2–3 days after the chambers were set up. Embryos were collected on grape-agar plates for a four-hour period, then transferred to fresh plates for accurate counting. At least 30 embryos were transferred per genotype per experiment. Embryos were incubated for an additional 24 h at 25 °C. The number of hatched and unhatched embryos were counted and embryo viability was calculated using the formula: (hatched embryos/total embryos) × 100. Statistical analysis via one-way ANOVA with multiple comparisons and graphing were performed using GraphPad Prism 10.5.0.

### 2.6. Ovary Dissection and Fixation

Virgin female flies of the desired genotype were collected and crossed with *w^1118^* males. Flies were maintained in a well-fed vial at 25 °C for three days before dissection. Ovaries were dissected in PBS and fixed in 3.7% formaldehyde for 20 min. Fixed ovaries were washed three times in PBS-Tx before proceeding to immunostaining.

### 2.7. Immunostaining

Fixed ovaries were incubated in primary antibody solution overnight at 4 °C on a rotating platform. The primary antibody solution was prepared with mouse anti-ɣH2aV (DSHB) at a concentration of 1:500 in PBS-Tx + 5% NGS. After washing three times in PBS-Tx, ovaries were incubated in secondary antibody solution for two hours at room temperature on a rotating platform. The secondary antibody solution was prepared with goat anti-mouse AlexaFluor488 (Thermo Fisher Scientific) at a concentration of 1:1000 in PBS-Tx. After the secondary antibody was removed, ovaries were incubated for two minutes in PBS-Tx + 1 µg/mL DAPI then washed twice in PBS-Tx. Stained ovaries were mounted in ProLong (Thermo). Imaging was performed using a Zeiss AxioObserver.Z1 with Apotome 2.0. For quantification of ɣH2aV staining, ten *w^1118^*, five *Dna2^lS/D1^*, and ten *Dna2^lS/D2^* germaria were assessed. Statistical analysis via Fisher’s exact test was performed using GraphPad Prism 10.5.0

### 2.8. Imaging and Quantification of Germarium Size

Imaging was performed using a Zeiss AxioObserver.Z1 with Apotome 2.0. Germaria were measured using the ZEN 3.5 software measurement tool. At least nine germaria were measured per genotype. Statistical analysis via one-way ANOVA with multiple comparisons and graphing were performed using GraphPad Prism 10.5.0.

### 2.9. Lifespan Assays

*Dna2^lS^/FM7c* females were crossed with *Dna2^D1^* or *Dna2^D2^* males. *w^1118^* were used as a wild type control instead of *Dna2^lS^/FM7c* non-mutants to mitigate balancer effects on lifespan and physiology. Progeny were allowed to mate for 24–48 h following eclosion and then *Dna2^lS/D1^* or *Dna2^lS/D2^* females were separated under CO_2_ anesthetization and placed into narrow vials. Flies were transferred to new food and dead flies counted every 2–3 days for the duration of the experiment. Three biological replicates were completed. All analyses accounted for experimental batch structure by stratifying on experiment. Initially, stratified Cox proportional hazards regression was attempted, but Schoenfeld residual tests revealed violations of the proportional hazards assumption (global test *p* < 0.05). Therefore, stratified log-rank tests were performed as our primary analysis, which provide robust comparisons of survival distributions without assuming constant hazard ratios. Stratified log-rank tests were performed using R statistical software (version 4.5.0) with the survival package [24,25,26]. Claude Sonnet 4 (Anthropic, San Francisco, CA, USA) was used for assistance with R code development for survival analysis and statistical methodology guidance for handling experimental batch effects. Graphs were made using GraphPad Prism 10.5.0.

## 3. Results

### 3.1. Dna2 Responds to Specific Types of DNA Damage

**Figure 1:**
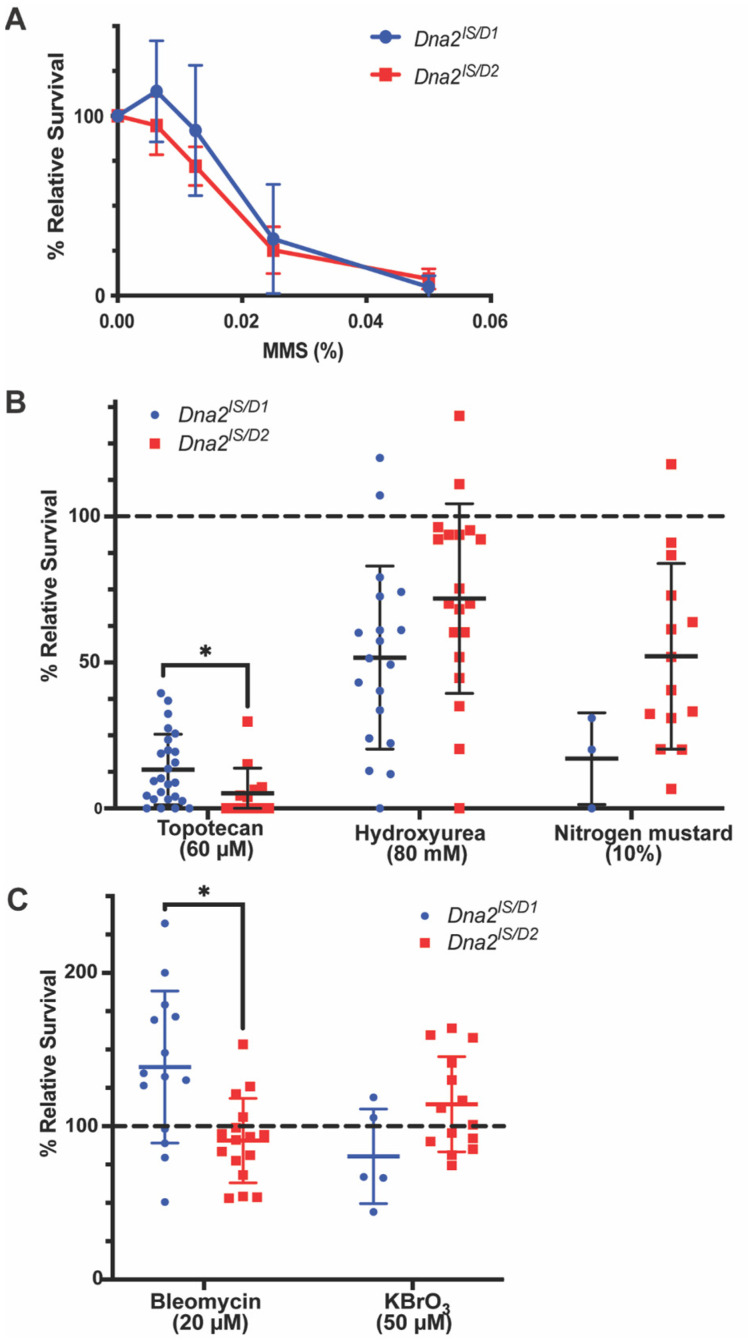
*Dna2* responds to replication stress, but not double-strand breaks or crosslinks. *Dna2* mutant and non-mutant larvae were treated with mutagens and adult relative survival was calculated. (**A**) No significant differences were found between the response of *Dna2^lS/D1^* and *Dna2^lS/D2^* to increasing doses of MMS. *p* = 0.2487 by two-way ANOVA. *n* = 5–14 vials/dose. (**B**) When treated with mutagens that cause replication stress, *Dna2* mutants show relative survival significantly below the no-sensitivity reference (100%; dotted line). (Topotecan: *Dna2^lS/D1^* and *Dna2^lS/D2^ p* < 0.0001; Hydroxyurea: *Dna2^lS/D1^ p* = 0.0028, *Dna2^lS/D2^ p* = 0.1407; Nitrogen mustard: *Dna2^lS/D1^ p* = 0.0003, *Dna2^lS/D2^ p* = 0.0045, all by unpaired t-test). *Dna2^lS/D2^* showed significantly lower survival to topotecan compared to *Dna2^D1^*. * *p* < 0.05 by unpaired *t*-test. *n* = 3–24 vials/mutagen. (**C**) *Dna2* mutants are not sensitive to bleomycin or potassium bromate (KBrO_3_). *Dna2^lS/D1^* mutants show significantly higher relative survival to bleomycin compared to 100% relative survival (dotted line) which may indicate resistance (*p* = 0.0319 by unpaired *t*-test). *Dna2^lS/D1^* also showed significantly higher survival to bleomycin compared to *Dna2^lS/D2^*. * *p* < 0.05 by unpaired *t*-test. *n* = 5–16 vials/mutagen. Error bars represent ± SD in all graphs.

We have previously confirmed sensitivity of all *Dna2* alleles in this study to 0.05% MMS, including slight, but non-significant, differences between alleles possibly due to the presence of helicase 1A in *Dna2^D1^* [14]. To further examine possible differences between the alleles, we exposed *Dna2* mutants to lower concentrations of MMS. *Dna2^lS/D1^* and *Dna2^lS/D2^* mutants responded similarly to every concentration of MMS tested, displaying no sensitivity at 0.00625% MMS and near total lethality at 0.05% MMS (Figure 1A). There was no difference in MMS sensitivity between *Dna2^lS/D1^* and *Dna2^lS/D2^* mutants (*p* = 0.25 for interaction by two-way ANOVA) suggesting that there is no difference in Dna2 function between the two alleles when responding to MMS-induced damage.

The role of Dna2 in responding to DNA damage was further explored by treating *Dna2* mutant and non-mutant heterozygous larvae with various mutagens that cause specific DNA damage and calculating relative survival to adulthood. Consistent with previous data in other models [7,10,27,28], *Drosophila Dna2* mutants show low relative survival when treated with mutagens that cause replication fork stalling or collapse: topotecan (topoisomerase I inhibitor), hydroxyurea (ribonucleotide reductase inhibitor; depletes pool of available dNTPs) and nitrogen mustard (alkylating agent; causes DNA crosslinks) compared with 100% survival indicative of no sensitivity (Figure 1B). In contrast, *Dna2* mutants were not sensitive to bleomycin (causes DSBs) or potassium bromate (causes strand breaks through oxidative stress). Relative survival for bleomycin-treated *Dna2^D1^* larvae was significantly higher than 100% survival, which may indicate resistance to bleomycin-induced damage (Figure 1C, *p* < 0.05). We also observed a modest, but significant difference in survival between the two *Dna2* alleles where *Dna2^lS/D1^* showed higher survival when treated with topotecan and bleomycin, possibly indicating a helicase-specific role in responding to certain types of damage.

### 3.2. Dna2 Mutants Exhibit Reduced Viability and Reproductive Fitness

**Figure 2:**
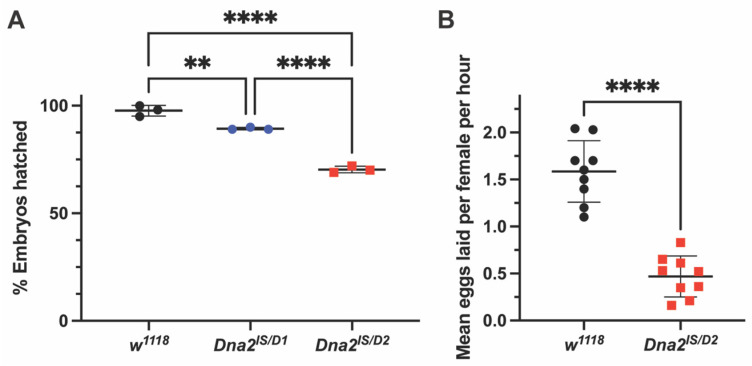
*Dna2* is required for normal fertility and fecundity. (**A**) Hatching frequencies were determined for eggs laid by *Dna2* mutants and *w^1118^* females. *n* = 3 experiments containing at least 30 embryos per experiment. ** *p* < 0.01 and **** *p* < 0.0001 by one-way ANOVA and multiple comparisons. (**B**) Eggs laid per female per hour by *Dna2* mutants and *w^1118^* females were determined by dividing the number of embryos collected by the number of females in the chamber and the length of the collection period (3 h). *n* = 9 embryo collections. **** *p* < 0.0001 by unpaired *t*-test. Error bars represent ± SD in all graphs.

*Dna2* mutants do not eclose in expected Mendelian ratios, even when factoring in the lethal *Dna2^lS^* allele. Offspring from the parental cross (*Dna2^lS^/FM7c* females × *Dna2^D1^/Y* or *Dna2^D2^/Y* males) produce significantly fewer non-mutant males (*FM7c/Y*) and more females than expected for both *Dna2^D1^* and *Dna2^D2^* crosses (Table 1, Χ^2^ *p* < 0.0001), which may be due to viability deficiencies with the *FM7c* balancer [29,30]. We next investigated the role of Dna2 in reproductive fitness by assessing mutant fertility and fecundity. Eggs produced by *Dna2* mutant females hatch at significantly lower rates than those produced by wild-type females, indicating reduced fertility (Figure 2A, *p* < 0.01 for *Dna2^lS/D1^* and *p* < 0.0001 for *Dna2^lS/D2^*). Hatching frequency of eggs laid by *Dna2^lS/D2^* females was also significantly lower than hatching frequency of eggs laid by *Dna2^lS/D1^* females, suggesting a difference in allele function (Figure 2A). A follow-up fecundity assay for the more severely affected *Dna2^lS/D2^* females revealed that *Dna2* mutants produce fewer eggs compared to *w^1118^* (Figure 2B, *p* < 0.001), suggesting a role for Dna2 in oogenesis.

### 3.3. Dna2 Is Required to Prevent Replication Stress in the Female Germline, Independent of Meiosis

**Figure 3:**
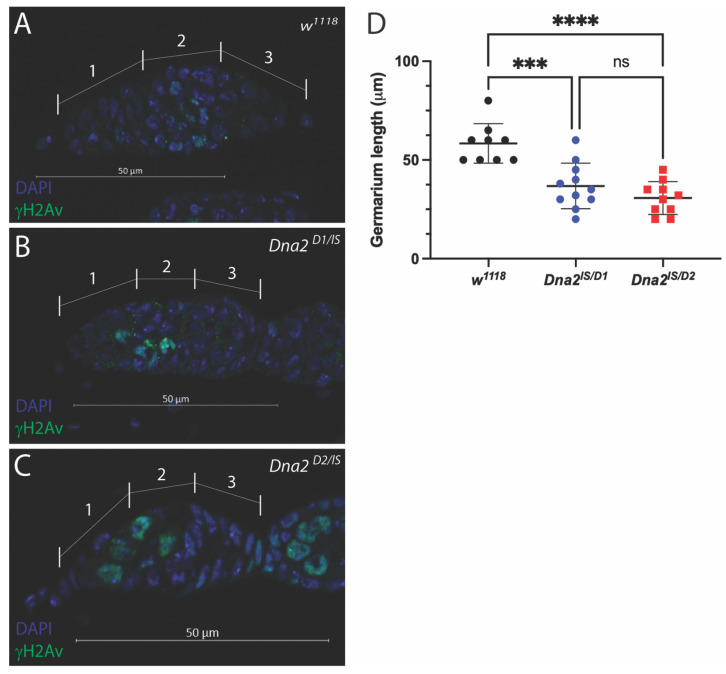
*Dna2* is not required for meiosis, but may prevent replication stress in the female germline. Germarium from (**A**) *w^1118^* female; (**B**) germarium from *Dna^lS/D1^* female; (**C**) germarium from *Dna^lS/D2^* female. Germaria stained for DAPI (blue) to label DNA and ɣH2aV (green) to label double-strand breaks. Germaria regions are labeled 1–3. Mitotically dividing germline stem cells and cystoblasts reside in region 1. Meiotic double-strand breaks are typically observed in region 2 but should be resolved before cysts progress into region 3. Note that, as expected, ɣH2aV-positive cells were observed in region 2 for all genotypes. Significantly more ɣH2aV-positive cells were observed in region 1 in *Dna2* mutants compared to *w^1118^* (*p* < 0.0001, *p =* 0.286 *w^1118^* vs. *Dna2^D1^*, and *p =* 0.0004 *w^1118^* vs. *Dna2^D2^* by Fisher’s exact test after Bonferroni correction). *n* = 5–10 germaria per genotype. (**D**) Germarium length was measured using the Zen program. *Dna2* mutant germaria were significantly smaller than *w^1118^* germaria. *n* = 9–11 germaria per genotype. *** *p* < 0.001, **** *p* < 0.0001, and ns = not significant by one-way ANOVA and multiple comparisons.

To better understand the cause of reduced fecundity and fertility in *Dna2* mutant females, we next examined ovarian morphology. We used immunostaining to label phosphorylated histone H2A variant (ɣH2Av), which is present during DNA damage and repair, and can also be used to visualize the resolution of induced double-strand breaks during meiosis [31,32,33,34,35]. Meiotic double-strand breaks are normally observed in regions 2A and 2B of the germarium, but should be absent in region 1, where germline stem cells and cystoblasts are undergoing mitotic divisions. Meiotic double-strand breaks should also be resolved by the time cysts transition into region 3 of the germarium [33]. As expected, we observed ɣH2Av-positive cells within regions 2A and 2B but not regions 1 or 3 of wild-type germaria, likely labeling cells undergoing meiosis I (Figure 3A). ɣH2Av-positive cells were also observed in regions 2A and 2B but not region 3 in *Dna2* mutant germaria, suggesting that meiosis proceeds normally in these mutants (Figure 3B,C). However, we sometimes observed ɣH2Av-positive cells in region 1 of *Dna2* mutant germaria (Figure 3C). We observed ɣH2Av-positive cells in region 1 in 40% of *Dna2^lS/D1^* germaria and in 90% of *Dna2^lS/D2^* germaria, which are significantly more than *w*^1118^ (*p* < 0.0001 by Fisher’s exact test). ɣH2Av staining in mitotically dividing cells in region 1 suggests DNA damage in germline stem cells and/or mitotically dividing cystoblasts. We also noted abnormal morphology of germaria and reduced overall size of mutant germaria (Figure 3C,D).

### 3.4. Dna2 Mutations Do Not Reduce Lifespan

**Figure 4:**
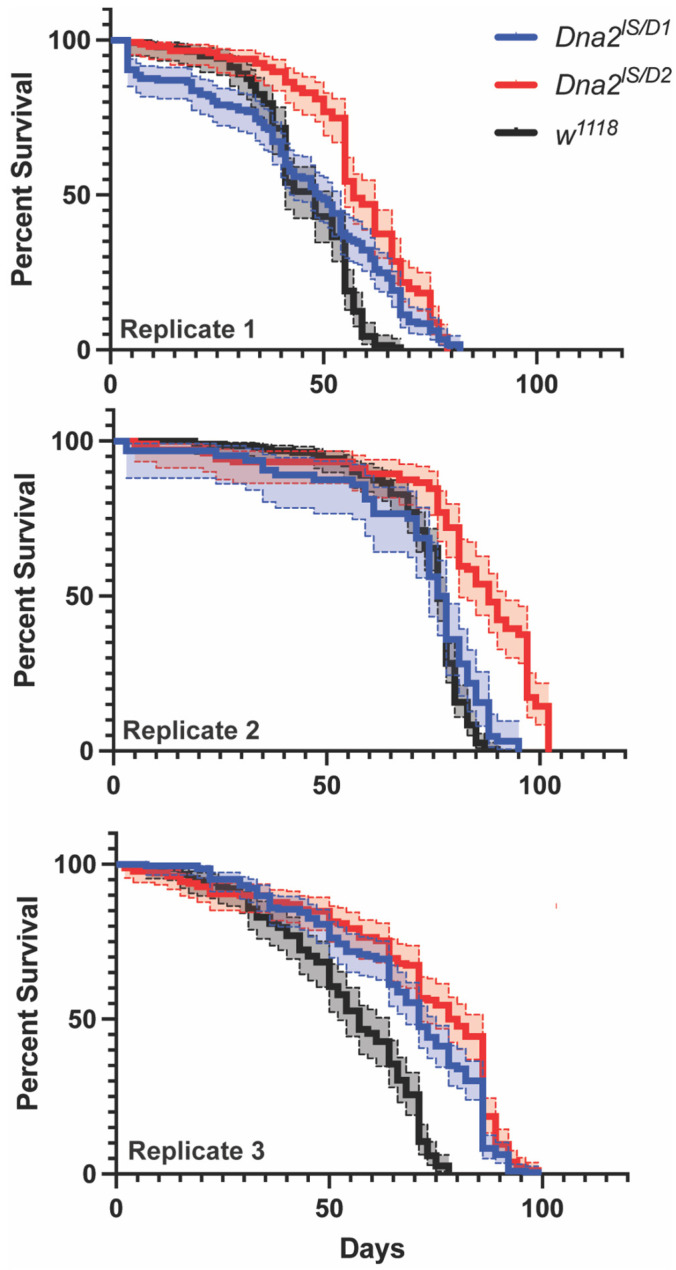
*Dna2* is not required for normal lifespan. All Kaplan–Meier survival curves differ from each other by genotype and experimental replicate (stratified log-rank test for overall comparison, Χ^2^ = 274.22, df = 2, *p* < 0.0001). *Dna2* mutants display longer lifespans compared to *w^1118^* (Χ^2^ = 308.54, df = 1, *p* < 0.0001), with longer lifespans in *Dna2^lS/D2^* flies compared to *Dna2^lS/D1^*. *n* = 140–181 (replicate 1), 64–190 (replicate 2), and 160–206 (replicate 3). (Χ^2^ = 51.08, df = 1, *p* < 0.0001). Shaded regions represent 95% confidence intervals.

We examined the effect of Dna2-deficiency in adults by measuring fly lifespan. There was great variability across our three biological replicates, with each genotype and replicate showing different survivorship curves for *w^1118^*, *Dna2^lS/D1^*, and *Dna2^lS/D2^* (Figure 4, *p* < 0.0001 by stratified log-rank analysis). Surprisingly, *Dna2* mutants showed longer lifespans compared to *w^1118^*, with *Dna2^lS/D2^* flies living longer than *Dna2^lS/D1^*.

## 4. Discussion

Proper DNA replication is critical for organismal survival, especially during early stages of development. Our study shows that Dna2 is required during oogenesis, as well as at critical developmental timepoints in early embryogenesis and metamorphosis. This requirement, which was assessed via loss-of-function analysis (sufficiency was not tested), is evidenced by reduced fecundity, fertility, and survival of larvae to adulthood following exposure to mutagens that induce replication stress. Additionally, we observed increased ɣ-H2Av in mitotically dividing regions in *Dna2* mutant germaria indicating that Dna2 may prevent damage in these tissues. Together, these findings indicate that Dna2 responds to stress during DNA replication in *Drosophila.*

Both the nuclease and helicase activity of Dna2 are critical for promoting replication fork recovery. The DNA2 nuclease removes 5′ RNA-DNA flaps during Okazaki fragment processing in coordination with the nuclease FEN1 [3,11]. DNA2 also promotes replication restart through degradation of reversed replication forks [36] and DNA end resection to initiate homology-directed repair of double-strand breaks along with RecQ helicases (reviewed in [3]), while protecting against excessive replication intermediates [5]. Evidence for essential helicase-dependent roles of Dna2 is more limited, showing that Dna2 supports homologous recombination (HR) in replication fork recovery in yeast [7] and at centromeric regions in human cells [37]. Interestingly, helicase activity is only strongly observed when nuclease activity is attenuated [8,38], further implicating the nuclease as the dominant enzymatic activity of Dna2. Our transheterozygous *Dna2* mutants contain one allele that lacks nuclease sequence (*Dna2^lS^*) and one allele that contains the nuclease (*Dna2^D1^* or *Dna2^D2^*). Despite the presence of one copy of the nuclease domain, these mutants are still hypersensitive to DNA damage that likely involves nuclease-dependent repair. This may suggest that the nuclease domain is compromised by improper protein folding in the *Dna2^D1^* and *Dna2^D2^* alleles, which will need to be investigated through biochemical analysis of the mutant alleles. The mutant alleles also differ in the presence of the helicase 1A domain where *Dna2^D1^* contains the helicase and *Dna2^D2^* does not. We observed modest, but significantly higher relative survival of *Dna2^lS/D1^* mutants treated with topotecan and bleomycin, possibly indicating helicase-dependent roles in responding to breaks at replication forks.

DNA2 responds to replication stress through restarting/resolving stalled replication forks or attending to DSBs caused by replication fork collapse. DNA2 has been shown to respond to DSBs in mitotic cells through its interactions with Exo1, MRN/X, and sgs1/Blm [12,39,40]. In the absence of DNA2, persistent replication intermediates accumulate, which can interfere with chromosome segregation and metaphase plate formation during mitosis [5,7,10,37,41]. DNA2 also responds to replication stress during meiosis [42], however the evidence is more limited due to redundancy with required proteins such as Exo1 and sgs1 [40]. Our data show that there is no increase in DSBs in the meiotically dividing cells in region 2 of *Dna2* mutant germaria. However, we observed anincrease in DSBs in mitotically dividing cells in region 1 of germaria indicating a Dna2 response to replication stress during mitosis only. Similarly, the low hatching frequency of *Dna2* mutant eggs is likely due to replication stress during syncytial division in early embryogenesis, similar to mutant phenotypes observed for other repair genes [22,43,44,45,46]. These differences in mechanism may be due to the inherent differences between mitotic and meiotic DSBs: meiotic DSBs are tightly regulated and processed by meiosis-specific nucleases and recombinases, in which Dna2 is less essential. Therefore, Dna2 may be limited predominantly to mitotic cells where it is required for end resection of breaks caused by replication fork collapse.

DNA2 has also been shown to participate in end-resection during DSB repair [12], specifically in replicating cells [10]. While likely not its primary mechanism, Dna2 may also be responding to DSBs caused by collapsed replication forks in *Drosophila*. Our evidence similarly suggests involvement of Dna2 in DSB repair based on induction of ɣH2Av foci in region 1 in the germaria and mutant sensitivity to ɣ-radiation [15,17]. Our *Dna2* mutants are also sensitive to topotecan which in doses used in this study, has been shown to induce sensitivity in HR-deficient flies, further indicating involvement in DSB repair [47]. Surprisingly, our *Dna2* mutants are not sensitive to the radiomimetic bleomycin, which is well-known to cause DSBs. The damage mechanism of bleomycin has been shown to be dose-dependent where low doses (<10.6 μM) incite more DSBs based on involvement with Rad52 and high doses (>10.6 μM) induce single-strand breaks (SSBs) based on involvement with Rad6 [48,49,50]. Due to differences in dosing and organism, it is difficult to make a direct comparison, however it is possible that our dose of 20 μM is inducing more SSBs, which are repaired by a Dna2-independent mechanism. It is also possible that DSBs are being induced and repaired by another mechanism in *Drosophila*, such as the end-resection proteins CtIP and Rif1 [51,52], as Dna2 is most active at replication forks in dividing cells and might not be an important player in canonical DSB repair.

In many cases, a mild amount of stress can elicit a positive protective response. For example, mild stress induction has been shown to extend lifespan in flies and *C. elegans* [53,54,55,56]. More specific to this work, flies lacking the DNA repair protein, WRNexo, show both increased antioxidant activity and thermal stress resistance, suggesting lack of WRNexo may heighten other stress responses [23]. Despite known roles of DNA2 in DSB repair and base excision repair [3], we observed surprisingly high survival rates of *Dna2* mutants when treated with both bleomycin, which causes DSBs, and potassium bromate, which causes oxidative stress. Additionally, *Dna2^lS/D1^* showed significantly higher than 100% relative survival, which may indicate potential resistance to bleomycin-induced damage. Therefore, while Dna2 may not respond directly to DSBs or oxidative DNA damage, Dna2-deficiency may heighten other protective mechanisms to allow greater survival when exposed to these exogenous stressors.

Heightened stress responses may also be a factor in differences in longevity in *Dna2* mutants. We had hypothesized that lack of Dna2 would reduce fly lifespan by limiting repair capacity. Instead, we found that *Dna2* mutants have significantly longer lifespans compared to wild-type flies in an allele-dependent manner. Interestingly, low fecundity has been observed in long-lived flies, suggesting a trade-off in resource allocation from reproduction to longevity [57]. Therefore, low fecundity in *Dna2* mutants could lower their reproductive cost leading to increased longevity. Still, we interpret these results with caution as it is rare for DNA repair deficiency to extend lifespan and we acknowledge that genetic background differences in our *w^1118^* controls as well our *Dna2* alleles may have contributed to our results.

Together our results support a requirement for *Drosophila* Dna2 in responding to replication stress, both caused by DNA damaging reagents and during developmental processes that require rapid DNA replication. These findings are consistent with those in other organisms and support using *Drosophila* as a model for studying Dna2 function. We expect that these findings will support future studies into the molecular roles of Dna2 in *Drosophila* and help build the foundation for understanding how this protein contributes to genome maintenance and disease.

## Figures and Tables

**Table 1:** Mendelian ratio analysis for *Dna2* mutants.

Genotype	Observed Mutant Females	Observed Non-Mutant Females	Observed Non-Mutant Males	Expected Count (All Classes)	X^2^	*p*-Value
*Dna2^lS/D1^*	4218	4341	3074	3877.667	251.797	<0.0001
*Dna2^lS/D2^*	5141	4918	3507	4522	349.236	<0.0001

## Data Availability

The raw data supporting the conclusions of this article will be made available by the authors on request.

## References

[1] Hoeijmakers J.H.J. (2009). DNA Damage, Aging, and Cancer. N. Engl. J. Med..

[2] Jackson S.P., Bartek J. (2009). The DNA-damage response in human biology and disease. Nature.

[3] Zheng L., Meng Y., Campbell J.L., Shen B. (2020). Multiple roles of DNA2 nuclease/helicase in DNA metabolism, genome stability and human diseases. Nucleic Acids Res..

[4] Appanah R., Jones D., Falquet B., Rass U. (2020). Limiting homologous recombination at stalled replication forks is essential for cell viability: DNA2 to the rescue. Curr. Genet..

[5] Falquet B., Ölmezer G., Enkner F., Klein D., Challa K., Appanah R., Gasser S.M., Rass U. (2020). Disease-associated DNA2 nuclease–helicase protects cells from lethal chromosome under-replication. Nucleic Acids Res..

[6] Hudson J.J.R., Rass U. (2021). DNA2 in Chromosome Stability and Cell Survival—Is It All about Replication Forks?. Int. J. Mol. Sci..

[7] Ölmezer G., Levikova M., Klein D., Falquet B., Fontana G.A., Cejka P., Rass U. (2016). Replication intermediates that escape Dna2 activity are processed by Holliday junction resolvase Yen1. Nat. Commun..

[8] Levikova M., Pinto C., Cejka P. (2017). The motor activity of DNA2 functions as an ssDNA translocase to promote DNA end resection. Genes Dev..

[9] Miller A.S., Daley J.M., Pham N.T., Niu H., Xue X., Ira G., Sung P. (2017). A novel role of the Dna2 translocase function in DNA break resection. Genes Dev..

[10] Peng G., Dai H., Zhang W., Hsieh H.J., Pan M.R., Park Y.Y., Tsai R.Y.-L., Bedrosian I., Lee J.-S., Ira G. (2012). Human Nuclease/Helicase DNA2 Alleviates Replication Stress by Promoting DNA End Resection. Cancer Res..

[11] Budd M.E., Choe W.-C., Campbell J.L. (2000). The Nuclease Activity of the Yeast Dna2 Protein, Which Is Related to the RecB-like Nucleases, Is Essential in Vivo. J. Biol. Chem..

[12] Pawłowska E., Szczepanska J., Blasiak J. (2017). DNA2—An Important Player in DNA Damage Response or Just Another DNA Maintenance Protein?. Int. J. Mol. Sci..

[13] Zhou C., Pourmal S., Pavletich N.P. (2015). Dna2 nuclease-helicase structure, mechanism and regulation by Rpa. eLife.

[14] Mitchell C., Becker V., DeLoach J., Nestore E., Bolterstein E., Kohl K.P. (2022). The Drosophila Mutagen-Sensitivity Gene mus109 Encodes DmDNA2. Genes.

[15] Boyd J.B., Golino M.D., Nguyen T.D., Green M.M. (1976). Isolation and Characterization of X-of Drosophila Melanogaster Which Are Sensitive to Mutagens. Genetics.

[16] Mason J.M., Green M.M., Shaw K.E., Boyd J.B. (1981). Genetic analysis of X-linked mutagen-sensitive mutants of Drosophila melanogaster. Mutat. Res..

[17] Nguyen T.D., Green M.M., Boyd J.B. (1978). Isolation of two X-linked mutants in *Drosophila melanogaster* which are sensitive to γ-rays. Mutat. Res. Mol. Mech. Mutagen..

[18] Beranek D.T. (1990). Distribution of methyl and ethyl adducts following alkylation with monofunctional alkylating agents. Mutat. Res. Mol. Mech. Mutagen..

[19] Povirk L.F., Shuker D.E. (1994). DNA damage and mutagenesis induced by nitrogen mustards. Mutat. Res..

[20] Azzam E.I., Jay-Gerin J.P., Pain D. (2012). Ionizing radiation-induced metabolic oxidative stress and prolonged cell injury. Cancer Lett..

[21] Schumacher B., Pothof J., Vijg J., Hoeijmakers J.H.J. (2021). The central role of DNA damage in the ageing process. Nature.

[22] Bolterstein E., Rivero R., Marquez M., McVey M. (2014). The Drosophila Werner Exonuclease Participates in an Exonuclease-Independent Response to Replication Stress. Genetics.

[23] Epiney D.G., Salameh C., Cassidy D., Zhou L.T., Kruithof J., Milutinović R., Andreani T.S., Schirmer A.E., Bolterstein E. (2021). Characterization of Stress Responses in a Drosophila Model of Werner Syndrome. Biomolecules.

[24] R Core Team (2020). R: A Language and Environment for Statistical Computing [Internet]. R Foundation for Statistical Computing. https://www.R-project.org/.

[25] Therneau T.M., Lumley T., Elizabeth A., Cynthia C. (2024). Survival: Survival Analysis in R, R Package.

[26] Therneau T.M., Grambsch P.M. (2000). Modeling Survival Data: Extending the Cox Model.

[27] Karanja K.K., Cox S.W., Duxin J.P., Stewart S.A., Campbell J.L. (2012). DNA2 and EXO1 in replication-coupled, homology-directed repair and in the interplay between HDR and the FA/BRCA network. Cell Cycle.

[28] Liu W., Zhou M., Li Z., Li H., Polaczek P., Dai H., Wu Q., Liu C., Karanja K.K., Popuri V. (2016). A Selective Small Molecule DNA2 Inhibitor for Sensitization of Human Cancer Cells to Chemotherapy. eBioMedicine.

[29] Miller D.E., Cook K.R., Yeganeh Kazemi N., Smith C.B., Cockrell A.J., Hawley R.S., Bergman C.M. (2016). Rare recombination events generate sequence diversity among balancer chromosomes in Drosophila melanogaster. Proc. Natl. Acad. Sci. USA.

[30] Miller D.E., Kahsai L., Buddika K., Dixon M.J., Kim B.Y., Calvi B.R., Sokol N.S., Hawley R.S., Cook K.R. (2020). Identification and Characterization of Breakpoints and Mutations on Drosophila melanogaster Balancer Chromosomes. G3 Genes|Genomes|Genet..

[31] Jang J.K., Sherizen D.E., Bhagat R., Manheim E.A., McKim K.S. (2003). Relationship of DNA double-strand breaks to synapsis in Drosophila. J. Cell Sci..

[32] Lake C.M., Holsclaw J.K., Bellendir S.P., Sekelsky J., Hawley R.S. (2013). The Development of a Monoclonal Antibody Recognizing the Drosophila melanogaster Phosphorylated Histone H2A Variant (-H2AV). G3 Genes|Genomes|Genet..

[33] Lake C.M., Hawley R.S. (2012). The Molecular Control of Meiotic Chromosomal Behavior: Events in Early Meiotic Prophase in Drosophila Oocytes. Annu. Rev. Physiol..

[34] Madigan J.P., Chotkowski H.L., Glaser R.L. (2002). DNA double-strand break-induced phosphorylation of Drosophila histone variant H2Av helps prevent radiation-induced apoptosis. Nucleic Acids Res..

[35] Mehrotra S., McKim K.S. (2006). Temporal Analysis of Meiotic DNA Double-Strand Break Formation and Repair in Drosophila Females. PLOS Genet..

[36] Thangavel S., Berti M., Levikova M., Pinto C., Gomathinayagam S., Vujanovic M., Zellweger R., Moore H., Lee E.H., Hendrickson E.A. (2015). DNA2 drives processing and restart of reversed replication forks in human cells. J. Cell Biol..

[37] Li Z., Liu B., Jin W., Wu X., Zhou M., Liu V.Z., Goel A., Shen Z., Zheng L., Shen B. (2018). hDNA2 nuclease/helicase promotes centromeric DNA replication and genome stability. EMBO J..

[38] Budd M.E., Choe W.C., Campbell J.L. (1995). DNA2 Encodes a DNA Helicase Essential for Replication of Eukaryotic Chromosomes. J. Biol. Chem..

[39] Bonetti D., Martina M., Clerici M., Lucchini G., Longhese M.P. (2009). Multiple Pathways Regulate 3′ Overhang Generation at *S. cerevisiae* Telomeres. Mol. Cell.

[40] Manfrini N., Guerini I., Citterio A., Lucchini G., Longhese M.P. (2010). Processing of Meiotic DNA Double Strand Breaks Requires Cyclin-dependent Kinase and Multiple Nucleases. J. Biol. Chem..

[41] Wawrousek K.E., Fortini B.K., Polaczek P., Chen L., Liu Q., Dunphy W.G., Campbell J.L. (2010). Xenopus DNA2 is a helicase/nuclease that is found in complexes with replication proteins And-1/Ctf4 and Mcm10 and DSB response proteins Nbs1 and ATM. Cell Cycle.

[42] Zhai B., Zhang S., Li B., Zhang J., Yang X., Tan Y., Wang Y., Tan T., Yang X., Chen B. (2023). Dna2 removes toxic ssDNA-RPA filaments generated from meiotic recombination-associated DNA synthesis. Nucleic Acids Res..

[43] McVey M., Andersen S.L., Broze Y., Sekelsky J. (2007). Multiple Functions of Drosophila BLM Helicase in Maintenance of Genome Stability. Genetics.

[44] Nakayama M., Yamaguchi S.-I., Sagisu Y., Sakurai H., Ito F., Kawasaki K. (2009). Loss of RecQ5 leads to spontaneous mitotic defects and chromosomal aberrations in *Drosophila melanogaster*. DNA Repair.

[45] Ruchert J.M., Brady M.M., McMahan S., Lacey K.J., Latta L.C., Sekelsky J., Stoffregen E.P. (2022). Blm helicase facilitates rapid replication of repetitive DNA sequences in early *Drosophila* development. Genetics.

[46] Sekelsky J. (2017). DNA Repair in Drosophila: Mutagens, Models, and Missing Genes. Genetics.

[47] Thomas A.M., Hui C., South A., McVey M. (2013). Common Variants of Drosophila melanogaster Cyp6d2 Cause Camptothecin Sensitivity and Synergize with Loss of Brca2. G3 Genes|Genomes|Genet..

[48] Karras G.I., Jentsch S. (2010). The RAD6 DNA Damage Tolerance Pathway Operates Uncoupled from the Replication Fork and Is Functional Beyond S Phase. Cell.

[49] Keszenman D.J., Salvo V.A., Nunes E. (1992). Effects of bleomycin on growth kinetics and survival of Saccharomyces cerevisiae: A model of repair pathways. J. Bacteriol..

[50] Lisby M., Rothstein R., Mortensen U.H. (2001). Rad52 forms DNA repair and recombination centers during S phase. Proc. Natl. Acad. Sci. USA.

[51] Thomas M.S., Pillai G.S., Butler M.A., Fernandez J., LaRocque J.R. (2024). The epistatic relationship of Drosophila melanogaster CtIP and Rif1 in homology-directed repair of DNA double-strand breaks. G3 Genes|Genomes|Genet..

[52] Yannuzzi I., Butler M.A., Fernandez J., LaRocque J.R. (2021). The Role of Drosophila CtIP in Homology-Directed Repair of DNA Double-Strand Breaks. Genes.

[53] Cypser J.R., Johnson T.E. (2002). Multiple stressors in Caenorhabditis elegans induce stress hormesis and extended longevity. J. Gerontol. A Biol. Sci. Med. Sci..

[54] Le Bourg E. (2011). A cold stress applied at various ages can increase resistance to heat and fungal infection in aged Drosophila melanogaster flies. Biogerontology.

[55] Wu D., Cypser J.R., Yashin A.I., Johnson T.E. (2009). Multiple mild heat-shocks decrease the Gompertz component of mortality in Caenorhabditis elegans. Exp. Gerontol..

[56] Zada D., Sela Y., Matosevich N., Monsonego A., Lerer-Goldshtein T., Nir Y., Appelbaum L. (2021). Parp1 promotes sleep, which enhances DNA repair in neurons. Mol. Cell.

[57] Flatt T. (2011). Survival costs of reproduction in *Drosophila*. Exp. Gerontol..

